# Supplementary material for: Experimental validation of a modeling framework for upconversion enhancement in 1D-photonic crystals
Source: Nat Commun. 2021 Jan 4;12:104. doi: 10.1038/s41467-020-20305-x (PMC7782824; doi:10.1038/s41467-020-20305-x)
Supplement: Supplementary file 1 — Supplementary Information [file 41467_2020_20305_MOESM1_ESM.pdf]

Supplementary information

Experimental validation of a modeling framework for upconversion enhancement in 1D-photonic crystals

Clarissa L. M. Hofmann[1,2,*], Stefan Fischer[3], Emil H. Eriksen[4], Benedikt Bläsi[1], Christian Reitz[5], Deniz Yazicioglu[1,6], Ian A. Howard[2,7], Bryce S. Richards[2,7] and Jan Christoph Goldschmidt[1]

[1] Fraunhofer Institute for Solar Energy Systems, Heidenhofstraße 2, 79110 Freiburg, Germany;
[2] Institute of Microstructure Technology (IMT), Karlsruhe Institute of Technology, Hermann-von-Helmholtz-Platz 1, 76344 Eggenstein-Leopoldshafen, Germany;
[3] Department of Materials Science and Engineering, Stanford University, 496 Lomita Mall, Stanford, CA 94305, USA;
[4] Department of Physics and Astronomy, Aarhus University, Ny Munkegade 120, DK-8000 Aarhus, Denmark;
[5] Institute of Nanotechnology (INT), Karlsruhe Nano Micro Facility, Karlsruhe Institute of Technology, Hermann-von-Helmholtz-Platz 1, 76344 Eggenstein-Leopoldshafen, Germany;
[6] Laboratory for Nanotechnology, Institute of Micro Systems Technology – IMTEK, University of Freiburg, Georges-Köhler-Allee 103, 79110 Freiburg, Germany;
[7] Light Technology Institute (LTI), Karlsruhe Institute of Technology, Engesserstrasse 13, 76131 Karlsruhe, Germany;
[*] Email: clarissa.hofmann@ise.fraunhofer.de

Supplementary Note 1: Optimization of active layers

Absorption of β-NaYF$_4$:25%Er^{3+}

In simulations, we model the upconversion process of β-NaYF$_4$:25%Er^{3+} inside the photonic structure. The model has been developed for a bulk upconverter material[1]. In Supplementary Figure 1, the normalized absorption of the bulk material, that serves as input in simulations, as well as of the upconverter nanoparticles used in experiment is plotted.

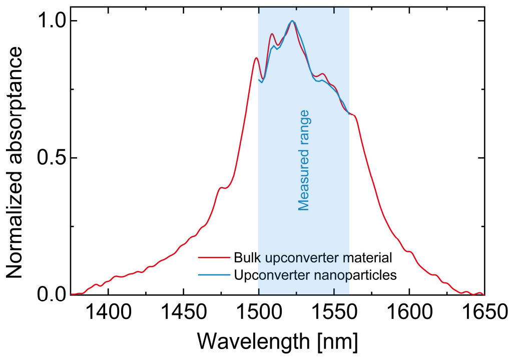

Supplementary Figure 1: Normalized absorptance of β-NaYF$_4$:25%Er^{3+} for the simulated bulk material and the upconverter nanoparticles used in experiment.

Spectrophotometer (Lambda 950, PerkinElmer, Germany) measurements were performed on the upconverter nanoparticles in toluene solution and in plain toluene. The normalized absorption of the upconverter nanoparticles was calculated from the transmittance and reflectance data of the upconverter nanoparticles, with plain toluene subtracted. One can see that the spectral dependence of both materials agree very well.

Production

The low refractive index layers of the Bragg structures are made of Poly(methyl methacrylate) (PMMA) (120,000 g/mol, purchased from Sigma-Aldrich), with embedded 25wt% of the purpose built core-shell upconverter nanoparticles β-NaYF$_4$:25% Er^{3+}. In the following, these layers are referred to as active layers. For the production, a stem solution of 10wt% PMMA in toluene and a stem solution of upconverter nanoparticles in toluene were mixed to give a final relation of 25wt% of upconverter nanoparticles with respect to the PMMA content, and 5wt% of PMMA with respect to the toluene content.

Thin layers were produced via spin-coating with a spin-coater SCS G3P-8 from Specialty Coating Systems (Alura Group BV). As a substrate, we used borosilicate glass (Borofloat 33, Schott AG) with a surface roughness smaller 1 nm. The substrates were cleaned for 15 minutes in aceton in an ultrasonic bath, then in isopropanol for 15 minutes in an ultrasonic bath. Directly prior to the spin-coating process, each substrate was dried with a nitrogen gun. 250 µL of solution were added within the first seconds of the spin-coating process with a total length of 60 s. The spin-speed was varied between 500 rpm and 2000 rpm for layer thickness adaption. Afterwards the samples were annealed at 40°C for 5 minutes.

Thickness adjustment and roughness

The surface quality, roughness and layer thickness, of the active layers were analyzed with an atomic force microscope (Dimension Edge, Bruker). The layer topography was measured with a scan rate of 0.7 Hz, a scan range of 2 µm with 512 lines in x and y direction for Supplementary Figure 2a. The measured roughness of the surface is 5.2 nm. The upconverter nanoparticles form islands of hexagonally organized monolayers that partly stick out of the PMMA layer surface. Apart from this roughness due to upconverter nanoparticles, the layer surface is very smooth.

The thickness adjustment of the active layers was done by fitting the thickness of layers produced with different spin speeds. The thickness measurements were performed by scratching a small cut into the layer and measuring the depth of the valley. The measurement was performed with a scan rate of 0.7 Hz, a scan range of 20 µm with 128 lines along the valley in x-direction and 1/16 of range and lines in y-direction. We measured a roughness on the layer surface of 8.5 ±1.1 nm for a 300 nm thin layer (mean and standard deviation of three measurements).

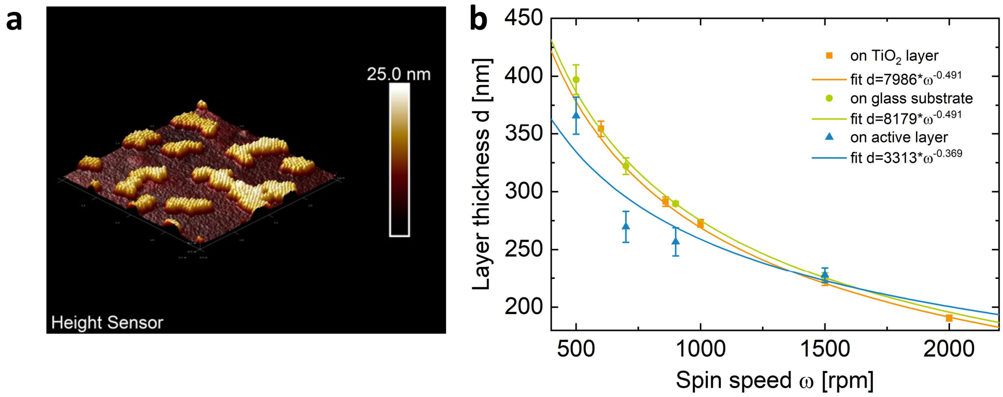

Supplementary Figure 2: Thickness adaption of active layers. a Topography of single active layer measured with an atomic force microscope, showing a small roughness of 5.2 nm due to upconverter nanoparticles at the layer surface for a 2 µm edge length. **b** Thickness adjustment in the spin-coating process of active layers on all substrate materials.

The depth was calculated as the difference between the mean of the area on the bottom of the valley and of the area on layer surface. It is important to include the surface roughness in the thickness adaption for a multilayer stack. Taking the mean of all heights worked out very well to gain an overall thickness control for our final production of multilayer stacks.

For various spin speeds, mean and standard deviation of the resulting thickness, out of 3 to 5 measurements for each spin speed were fitted. We use an empiric model, developed in[2] to link the coating thickness d to the spin speed ω and the concentration C as

$$d = D \cdot C^{\alpha} \cdot \omega^{\beta}, \qquad (1)$$

with the empirically determined parameters D, α and β. For our purpose, it is sufficient to fit the parameters $D \cdot C^{\alpha}$ as one variable. The thickness of a spin-coated layer significantly depends on the adhesion to the substrate material. For the production of a Bragg structure, we therefore performed the adaption on a TiO_2 layer. For the production of reference samples, the first active layer is spin-coated directly on the glass substrate, the following three active layers are spin-coated right on top of the other active layers. We therefore did the thickness adaption for each substrate, TiO_2, glass and an active layer, separately, as shown in Supplementary Figure 2b. The mean of all relative standard deviations of 18 measurements of the active layer thickness on TiO_2 is 1.3%, corresponding to 4.15 nm for a 315 nm thin layer. In simulations, we include this variation of the layer thickness.

Refractive index

We determined the refractive index of the thin active layers, as well as the refractive index and thickness of the thin TiO_2 layers on a silicon substrate via spectral ellipsometry. The used ellipsometer (M-2000, J.A.Woollam Co.) covers the wavelength range 245 nm to 1700 nm. We performed the ellipsometry measurement at the angles of 65° to 80° in steps of 5°. For fitting the data we used the software Complete Ease[3]. We chose to fit the active layer with a simple absorbing Cauchy model with a surface roughness[3]. For the wavelengths 1700 nm to 2000 nm, the refractive index data was extrapolated using a Cauchy model. This polymer-nanoparticle composite material with various low absorbing spectral regions is very difficult to fit and a more complex model did neither improve the quality of the fit nor the reliability of the result. The roughness thickness was in the range of 5 nm, slightly less but in agreement with the roughness determined in AFM measurements. With this model, we found a good agreement of fit and measurement, both thickness and real refractive index were well determined with a mean square error of 13.6 for a 241.6 nm thin layer. We assume that the determined real refractive index n is very precise, with a value of 1.474 at 1523 nm, shown in Supplementary Figure 3.

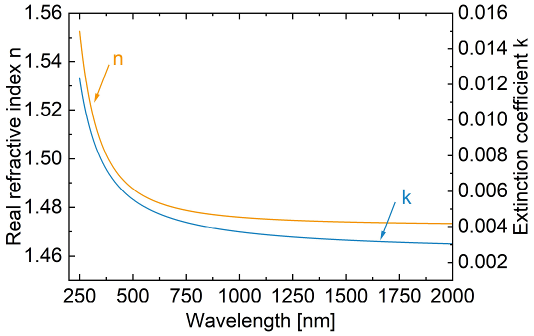

Supplementary Figure 3: Refractive index and extinction coefficient of the active layer material, PMMA with embedded upconverter nanoparticles.

However, the extinction coefficient cannot be determined precisely with this model. The absorption is very low and has a complex pattern with various absorbing regions within the upconverter material. Also from photospectrometer measurements, we know that the absorptance of the utilized upconverter nanoparticles is quite low, even in the main region of interest around 1523 nm. In fact, we could not precisely determine its value. Considering the absorption coefficient of a bulk upconverter material[1], we estimate the absorptance of one active layer to be below 0.01%. Therefore, as input in simulations, we only use the real refractive index and neglect the absorption, setting k to 0 for the simulation of the energy density within the Bragg structure. For a later application, however, this means that the number of layers and the loading of UCNP in the layer would need to increase.

Supplementary Note 2: Optimization of TiO_2 layers

Production

We use TiO_2 as high refractive index layer of the Bragg structure. The TiO_2 layers are produced with atomic layer deposition (R-200 Advanced, Picosun, Finland) from molecular precursors $TiCl_4$ (purchased from Sigma-Aldrich (≥ 99% $TiCl_4$)) and H_2O. To be compatible with PMMA, we used a low temperature process at 100°C. In the deposition process, rinsing and pulse times were: 0.1 s $TiCl_4$ pulse, 4 s rinsing with Ar, 0.1 s H_2O pulse, 6 s rinsing with Ar. The total number of pulses was 4000 to gain a layer thickness of around 200 nm.

Crystallinity

The crystallinity of the thin TiO_2 films was determined in X-ray diffraction (XRD) measurements (XRD D8, Bruker, equipped with an x-ray source of Cu-anode with a Cu $K\alpha_{1,2}$ radiation (λ = 0.15419 nm). We performed measurements in the range of 10° to 80° in steps of 0.05°. Supplementary Figure 4 shows the measurement of a plain glass substrate (*i*), glass with one active layer (*ii*), glass with one TiO_2 layer (*iii*) and a Bragg structure with 4 layers of TiO_2 and 3 active layers (*iv*).

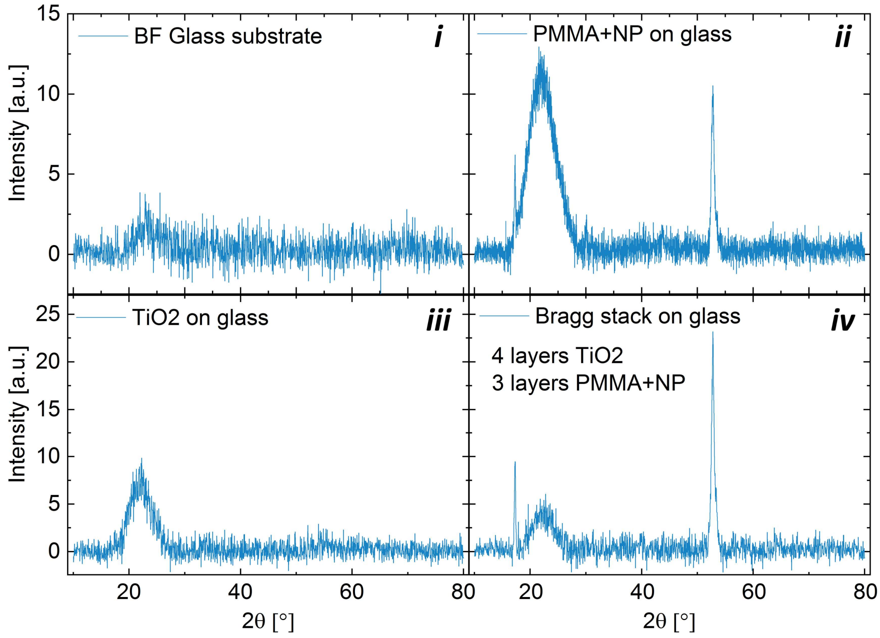

Supplementary Figure 4: Determination of TiO_2 crystalinity. X-ray diffraction measurements of only a glass substrate (***i***), one active layer on glass (***ii***), one TiO_2 layer on glass (***iii***) and a Bragg structure made up of four TiO_2 layers and three intermediate active layers on glass (***iv***).

For anatase TiO_2, usually forming at temperatures above 200°C, we mainly expect to see a reflection on the A101 bulk anatase plane around 25.5°[4–8]. Both, the single TiO_2 layer (c) and the Bragg structure (d) do not depict this reflection and the TiO_2 films are amorphous, as expected for our low deposition temperature. Compared to graph (a) and (b) we can conclude that the appearing broad and small peaks, also visible in graphs (c) and (d), stem from the glass substrate and active layer.

Refractive index and thickness determination

We performed spectroscopic ellipsometry measurements on thin TiO_2 layers on a silicon substrate the same way as described in the Supplementary Note 1. To fit the data, we applied a Cody-Lorentz model[3], as it describes amorphous materials with a broad Lorentzian absorption. In the model, we included a surface roughness that was fitted to 1 nm to 2 nm. The fit describes the measured data very well with a mean square error of 6.0 for a 202.0 nm thin TiO_2 film. Supplementary Figure 5 shows the fitted refractive index, with a real refractive index n of 2.279 and extinction coefficient k of 0 at 1523 nm wavelength.

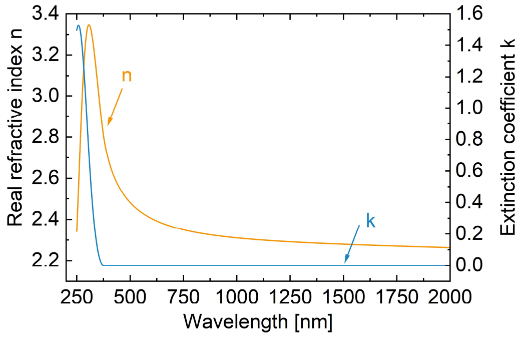

Supplementary Figure 5: Refractive index and extinction coefficient of TiO_2.

The model allows for a very precise fit of both refractive index and layer thickness. Therefore, we used this method to adapt the TiO_2 layer thickness for Bragg structure production.

Layer thickness adaption

The layer thickness of TiO_2 was determined in spectroscopic ellipsometry measurements. In the ALD chamber, we placed 30 glass substrates of 25 mm times 25 mm size on an 8 inch silicon wafer (Supplementary Figure 6).

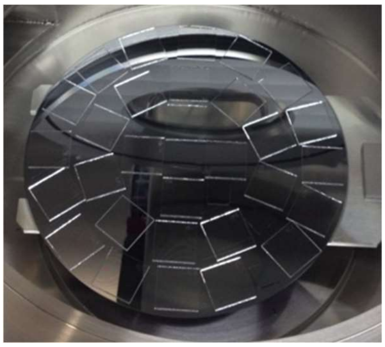

Supplementary Figure 6: Sample positioning in atomic layer deposition chamber for production of TiO_2 layers.

We analyzed the resulting thickness of the TiO_2 layer in one deposition run at two points b and d on each sample (Supplementary Figure 7a). We found that within the deposition chamber, the TiO_2 layer thickness varied slightly in the range from 189 nm to 213 nm. This stable inhomogeneity was favourable for us because it allowed for a controlled production of different sample designs in one deposition run.

We analyzed the standard deviation of the thickness of the produced TiO_2 layers with ellipsometry measurements on the silicon substrate wafer. For each deposition run (except the first), we used a new silicon substrate wafer. Therefore, we measured at one distinct point in the deposition chamber on all 4 substrate wafers and calculated the mean and standard deviation of these 4 points. We analyzed 16 points separately and subsequently calculated the mean of all standard deviations. This mean standard deviation of 1.53 nm, corresponding to 0.8%, calculated from 64 measurements in total, served as input in simulations for the standard deviation of the Gaussian distribution in the layer thickness variation.

Supplementary Note 3: Optimized Bragg structures

Design wavelength determination

On each fabricated Bragg structure sample we characterized five distinct points, as shown in Supplementary Figure 7a.

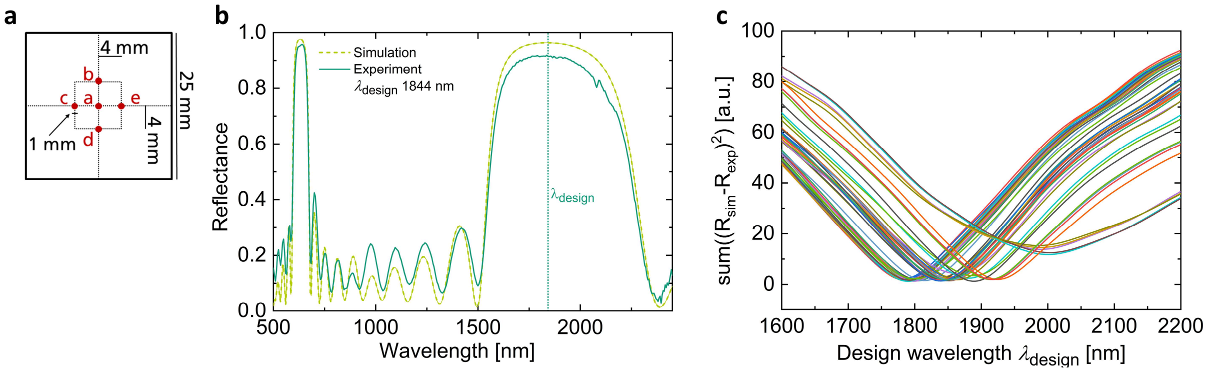

Supplementary Figure 7: Determination of design wavelength of Bragg structure via reflectance analysis. **a** Sample dimensions and measurement areas of the five small analyzed points a-e. **b** Exemplary matched simulated and measured reflectance. **c** Mean square difference between simulated and measured reflectance for the determination of design wavelength of each sample and measured points a-e.

The design wavelength slightly varies throughout each sample. The evaluated small measurement spot allows for a precise determination of the design wavelength, via the characteristic reflectance of a Bragg structure. We measured the reflectance with a spectrophotometer (Lambda 950, PerkinElmer, Germany) from 500 nm to 2445 nm with a step width of 5 nm. The incident light beam of the spectrograph was reduced with an aperture to feature a spot of approximately 1 mm diameter on the sample. We performed the measurement in an integrating sphere with a tilt of the sample of 8°.

We simulated the Bragg structure reflectance in an implementation of the transfer matrix method[9]. The determined refractive indices served as input parameters, as well as the details from experiment of angle and wavelength range. Supplementary Figure 7b shows the simulated reflectance for an exemplary Bragg structure with a determined design wavelength $\lambda_{\mathrm{design}}$ = 1844 nm. The position of the reflectance peak and side lobes in the measured and simulated reflectance are in good agreement. From this agreement, we can conclude that the fabricated Bragg structure is indeed very close to the target design. However, there are two slight deviations visible between simulation and experiment. One is the deviation of the side lobes, which we expect to stem from slight deviations in the single layer thicknesses in experiment due to small production inaccuracies. The other is the height of the main reflectance peak, which we expect to be due to the choice of white standard in the reflectance measurement. However, this feature is not relevant for our investigation. We analyzed $\lambda_{\mathrm{design}}$ of each measured point by the minimum squared difference of the measured reflectance R_{exp} to simulated reflectance curves $R_{\mathrm{sim}}(\lambda_{\mathrm{design}})$ with a binning of 1 nm in $\lambda_{\mathrm{design}}$

$$\mathrm{minimum}_R = \min\left(\sum\left(R_{\mathrm{exp}} - R_{\mathrm{sim}}\right)^2\right). \tag{2}$$

$\lambda_{\mathrm{design}}$ of the measured reflectance $\lambda_{\mathrm{design}}(R_{\mathrm{exp}})$ is then given by

$$\lambda_{\mathrm{design}}(R_{\mathrm{exp}}) = \lambda_{\mathrm{design}}(\mathrm{minimum}_R) \tag{3}$$

Supplementary Figure 7c shows the sum squared difference for all analyzed sample points. All curves show a well-defined minimum that clearly determines $\lambda_{\mathrm{design}}(R_{\mathrm{exp}})$. As can be seen, the sample points with $\lambda_{\mathrm{design}}$ around 2000 nm depict the largest deviation from simulation. This is due to one layer showing a quite large difference to the target thickness due to a slight instability in the spin-coating process for this layer. However, the overall design wavelength of these Bragg structure can be clearly defined.

Uniformity, nanoparticle distribution and roughness

We analyzed Bragg structure cross sections with a scanning electron microscope (Auriga 60, Zeiss). In the measurement, we used an in-lens detector and 1 kV voltage of the electron beam EHT. We broke the samples mechanically and sputtered them with a thin 3 nm platinum film. In the scanning electron microscopy (SEM) image of a Bragg structure cross section (Fig. 1b), the different constituents as well as the precision of production, layer uniformity and smoothness can be seen very clearly. The upconverter nanoparticles form small clusters and some are positioned at the active layer surface. A reason for this behaviour could be the polymerisation when the layer dries out during the spin-coating process. However, the clusters are small enough to not influence the layer uniformity. The upconverter nanoparticles at the layer surface represent the roughness that we included in the layer thickness adaption and do not significantly influence the overall Bragg structure design. Overlaying the SEM image with a Bragg structure with the target ratio of low- and high refractive index layer, shows that the relation between the high- and low refractive index layers is very close to the target relation, which again is a proof of quality. In the PMMA layers, fringes are visible, which stem from the degradation of PMMA under the electron beam. Supplementary Figure 8a displays a larger area, showing the very high uniformity also in a larger scale.

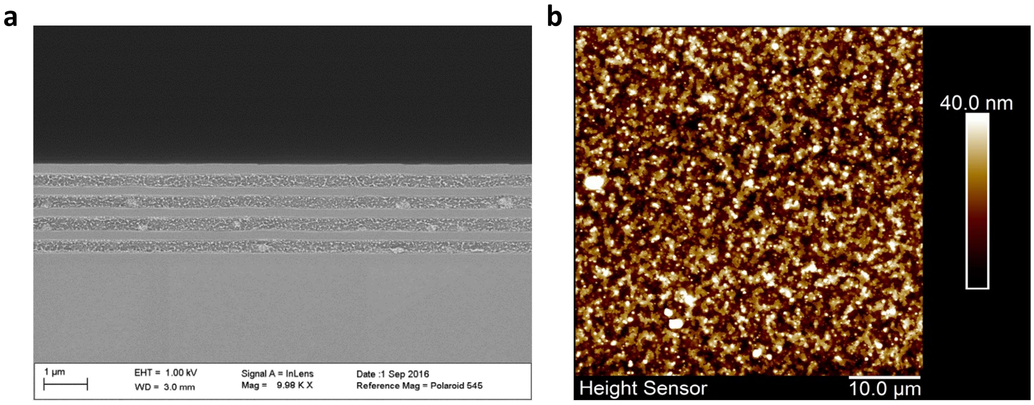

Supplementary Figure 8: Layer uniformity of a Bragg structure. a Scanning electron microscopy image of a large Bragg structure cross section, demonstrating the high uniformity. **b** Atomic force microscopy scan on the Bragg structure surface roughness.

Finally, we investigated how the roughness of each active layer influences the overall roughness of a complete Bragg structure. The SEM images show that the roughness of one active layer evens out throughout the Bragg structure latest with the next active layer. A large area atomic force microscopy scan (using the device Dimension Edge, Bruker) (Supplementary Figure 8b) confirms this observation. The monolayers of hexagonally organised islands of upconverter nanoparticles are still visible. There are some clusters visible, higher than one monolayer. However, the roughness of a complete Bragg structure, here measured as 10.1 nm, is not significantly higher than the roughness of one single active layer of up to 8.5 nm (compare to Supplementary Note 1). The image was taken for a 50 µm scan range with 1024 lines in x- and y direction and a scan rate of 0.2 Hz.

Supplementary Note 4: Upconversion photoluminescence

Photoluminescence measurement setup

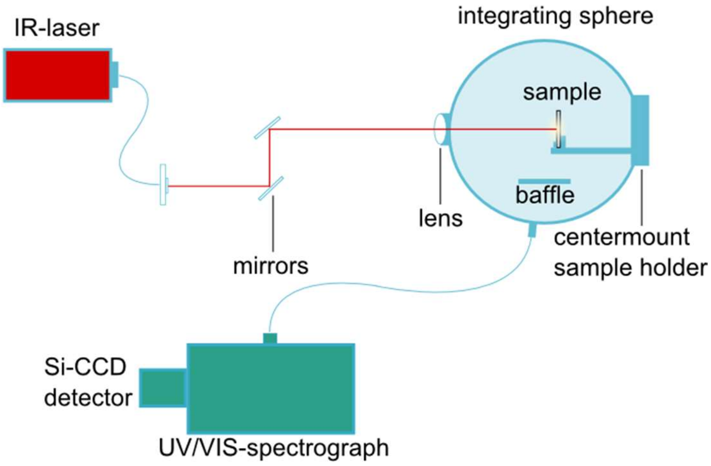

Supplementary Figure 9: Sketch of photoluminescence measurement setup.

Irradiance determination

For the determination of the laser irradiance, we measured the area of the laser beam with a beam profiler (BP209-IR/M, Thorlabs), the laser power with a photodiode sensor (PD300-IR, Ophir Photonics). Supplementary Figure 10a, shows the Gaussian-shaped beam profile at 1.48 W cm^{-2} irradiance and 1523 nm excitation wavelength.

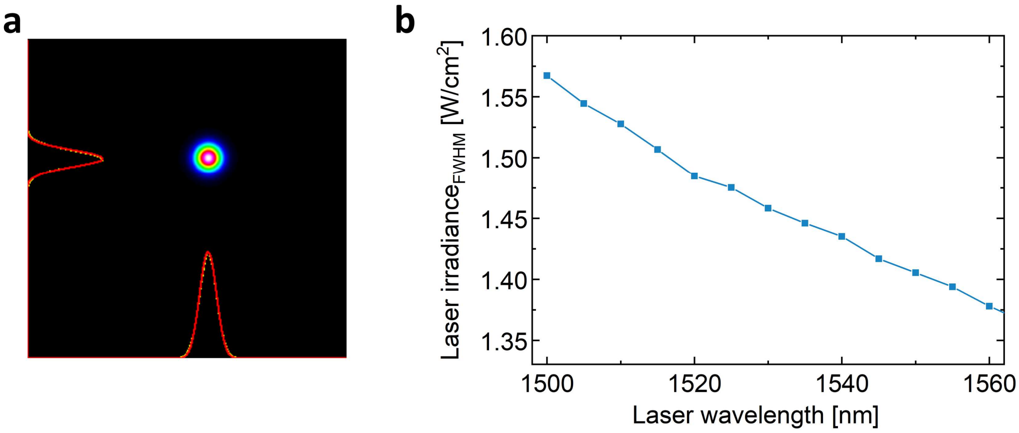

Supplementary Figure 10: Determination of laser irradiance. a Laser beam profile. **b** Determined laser irradiance for varied laser wavelength.

The difference of diameter in x- and y-direction of the Gaussian profile of all measurements is smaller 0.7%. We decide to calculate the laser irradiance only from the FWHM region. For the laser area, this region was given directly as output. Supplementary Figure 10a displays a FWHM area of 0.27 mm^2, corresponding to a diameter of 0.59 mm. The diameter slightly varies for a varied power and excitation wavelength in the range of 0.57 mm and 0.61 mm. We scaled the total laser power P_{total} to the fraction of power P_{FWHM} below the FWHM region, given by:

$$P_{\mathrm{FWHM}} = P_{\mathrm{total}} \cdot 0.5 \tag{4}$$

Additionally scaling the area by the tilt of the samples of 4°, we then calculated the irradiance as follows:

$$\text{Irradiance}_{\text{FWHM}} = \frac{P_{\text{FWHM}}}{A_{\text{FWHM}}/\cos(4°)} \tag{5}$$

We determined the irradiance at 1523 nm excitation wavelength for the power range 1 mW to 10 mW in steps of 0.5 mW as well as for an excitation power of 10 mW and the excitation wavelength range 1500 nm to 1560 nm in steps of 5 nm (Supplementary Figure 10b). All needed values between these measurements we interpolated.

Simulation of UCPL and UCQY

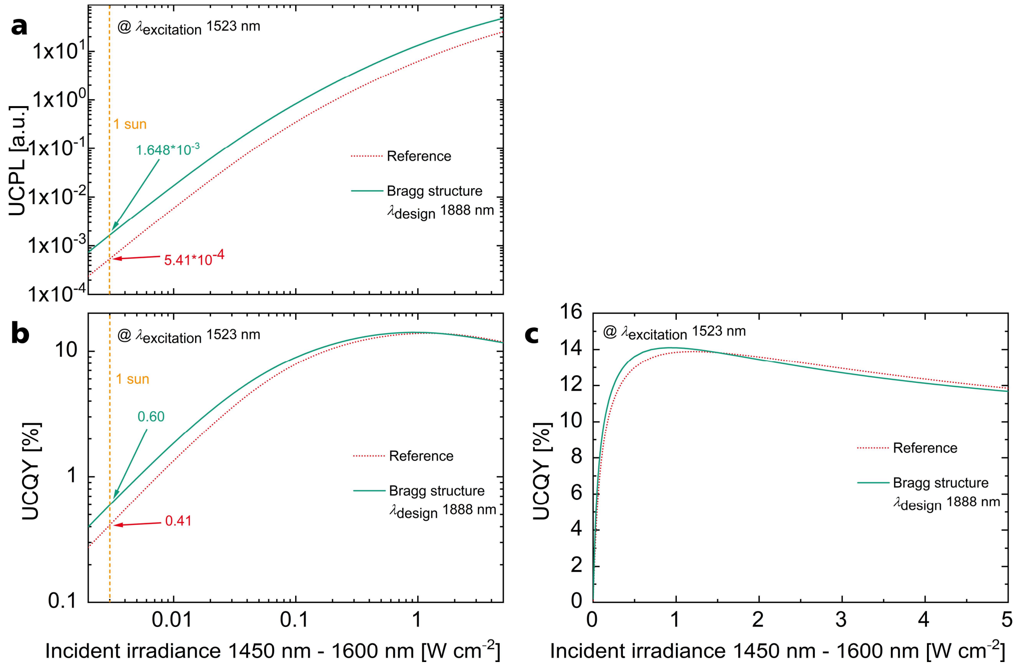

Supplementary Figure 11: Simulated upconversion (UC) efficiency down to 1 sun irradiance, at an excitation wavelength of 1523 nm for reference and Bragg structure design as in Fig. 4c of the manuscript. a, UC photoluminescence (UCPL). **b**, internal UC quantum yield (UCQY) on a logarithmic scale. **c**, UCQY on a linear scale.

Supplementary Figure 11a shows the simulated UC photoluminescence (UCPL) and internal UC quantum yield (UCQY) of the main UC emission at 984 nm down to one sun irradiance. The simulated Bragg structure design is equivalent to the design for which the dependence of the relative UCPL on the irradiance is shown in Fig. 4c in the manuscript. Supplementary Figure 11a and Supplementary Figure 11b show the UCPL and UCQY, respectively, on a logarithmic scale, to visualize the lower irradiance regime, while in Supplementary Figure 11c, the UCQY is plotted on a linear scale, as mostly done in literature. The internal UCQY is defined as the number of emitted photons in the UC emission at 984 nm per absorbed photon[9].

The UCPL increases with higher incident irradiances, such that the Bragg structure always outperforms the reference. The UCQY depicts a maximum. At irradiances above this maximum, other UC emissions become more likely that require the energy of more than two photons to participate in the UC process. This leads to a decrease in the UCQY of the 984 nm UC emission. Going towards lower irradiances, the photonic effects of the Bragg structure become exceedingly important compared to the reference. At one sun irradiance, the UCQY in the Bragg structure reaches 0.60%, while the reference only reaches 0.41%.

Higher order upconversion emission

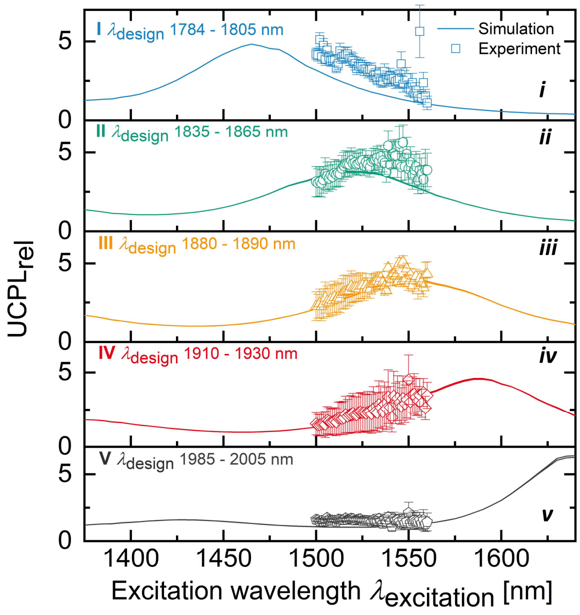

Supplementary Figure 12 Comparison of simulation and experiment of the relative upconversion photoluminescence ($UCPL_{rel}$) around 814 nm of the $^4I_{9/2}$ to $^4I_{15/2}$ transition. In a scan of the excitation wavelength $\lambda_{excitation}$, five groups of similar design wavelength λ_{design} are analyzed: at the expected maximum effect of the photonic structure on upconversion at λ_{design} = 1855 nm (***ii***), as well as around the maximum effect (***i, iii, iv***) and in the expected region of a suppressed $UCPL_{rel}$ (***v***). The absolute enhancement factors as well as the slope of all curves show good agreement in simulation and experiment. Source data are provided as a Source Data file.

Varied incident angle

For UC photoluminescence measurements with a varied incident angle we used the same excitation and detection device as described in the methods Section 4.2 of the manuscript. A 25.4 mm focal length lense was installed 110 mm in front of the sample to avoid unwanted oscillations in the glass substrate. We varied the excitation angle from 0° to 75° in steps of 5° and the excitation wavelength from 1500 nm to 1560 nm in steps of 1 nm. Detection is performed at 18° relative to the surface normal of the sample, while the signal is coupled in for an opening half cone of 7°. The data is again corrected with a spectral response correction function measured with the same units as described in Section 4.2.

From the laser characterization, we calculate the FWHM irradiance at 1523 nm excitation wavelength, including the beam widening due to the lense, on the sample surface to 0.2546 W cm^{-2}.

We correct both simulated and measured data such that the output of both features the same conditions: The simulation is performed for a fix excited sample volume, independent of the incident angle θ. Hence, we normalize the measured signal $S_{measured}(\theta)$ to the same area and light path as at 0° incidence, yielding a normalized signal $S_{normalized}(\theta)$ of

$$S_{normalized}(\theta) = S_{measured}(\theta) \cdot \cos^2(\theta). \quad (6)$$

In experiment, the irradiance at 0° incidence $I(0°)$ decreases by a factor of $\cos(\theta)$ for a varied incident angle θ. Due to the non-linear dependence of UC on the irradiance, this needs to be adapted likewise in simulation. The simulation is performed at $I(0°) = 0.2546$ W cm^{-2} irradiance and

$$I(\theta) = I(0°) \cdot \cos(\theta), \quad (7)$$

for θ of 0° to 75° in steps of 1°. The excitation wavelength is varied from 1500 nm to 1560 nm in steps of 1 nm, all with the same irradiance stated above. The slight difference in irradiance in experiment by approximately +6% at 1500 nm and -7% at 1560 nm is neglected in simulation. The simulation is performed with the same methods as described in Sections 3.8, 3.9 and 3.10 in the manuscript, in this case for an ideal Bragg structure, neglecting

production inaccuracies. However, from Figure 3b, we know that this neglect has only little impact on the simulation result. To match the experiment, the last important step is to include the directionality of emission in the simulation. However, introducing the methods is out of the scope of this paper and subject to our future work. Here, the simulation features the integrated emission into all angles. The fraction of emitted light that is coupled out of the Bragg structure into a specific detection angle and detection cone is different than the fraction of emitted light coupled out of the reference structure into the same detection angle and cone. This difference needs to be included when comparing the enhancement factors appearing in Bragg structure and reference. In our measurements, the detection angle is the same for all investigated incident angles. Therefore, the correction factor is a constant for all incident angles within the Bragg structure measurements and it is a different constant for all incident angles within the reference structure measurements. Consequently, when calculating the relative UCPL ($\mathrm{UCPL_{rel}}$) the correction factors only change the enhancement factors that are reached. The trends are not effected and can therefore be compared.

Supplementary Figure 13 shows the simulated reflectance (a, b), as well as the simulated (c, d) and measured (e) UC photoluminescence ($\mathrm{UCPL_{rel}}$) for three experimentally investigated sample designs (*i-iii*), demonstrating that light can efficiently be coupled in from a large angle range up to around 30°, which is a highly relevant factor for photovoltaic applications. Furthermore, the three different designs (i-iii) show that both the spectral and angle range, that is efficiently coupled in, can be tuned by choosing a suitable design wavelength for a particular target application.

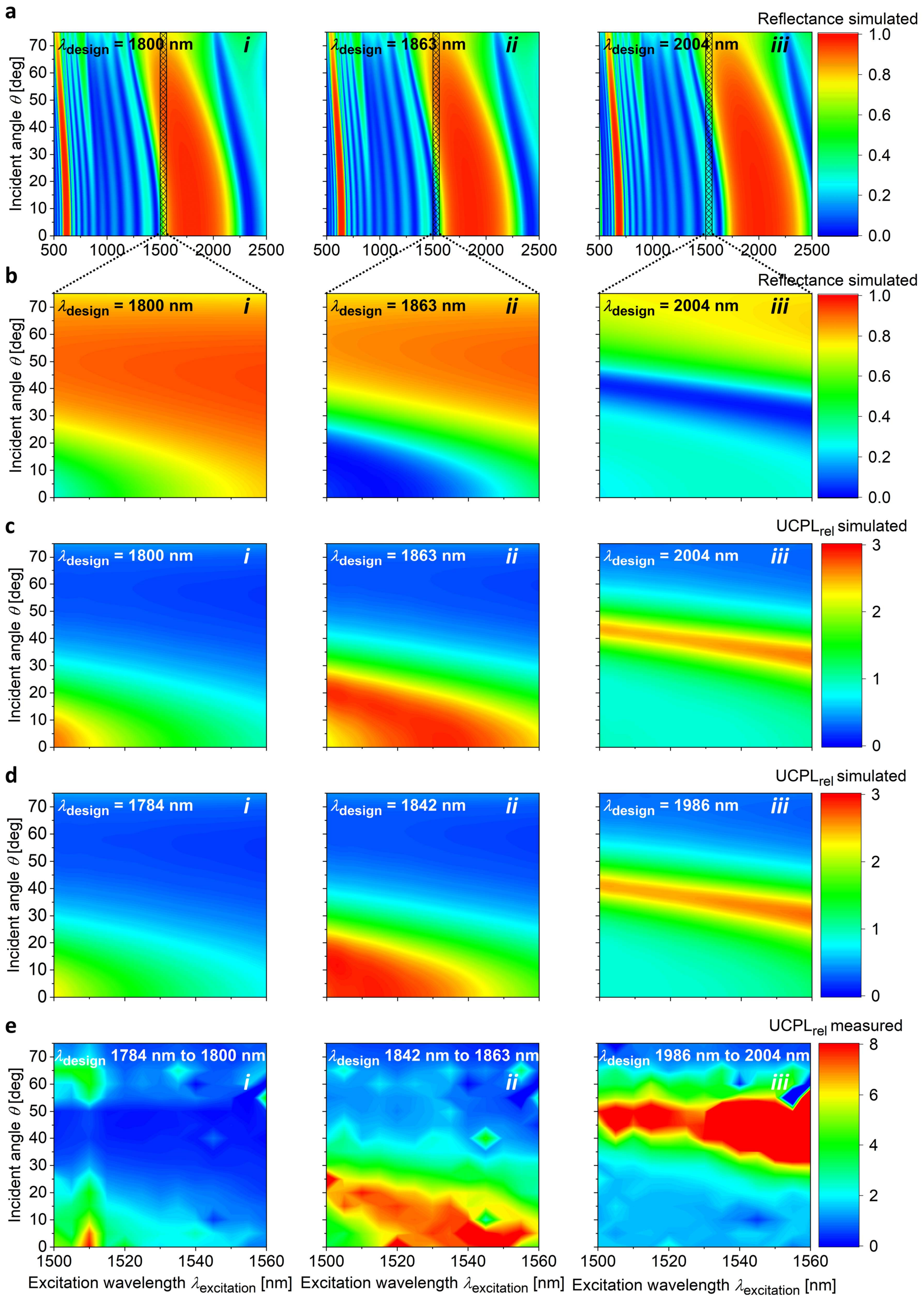

Supplementary Figure 13: Investigation of the relative upconversion photoluminescence (UCPL$_{rel}$) in different Bragg structure designs for a spectrally and angle resolved excitation. a, simulated reflectance for three different experimentally investigated Bragg structure designs to illustrate the shift of the photonic bandgap. The maximum design wavelengths measured on each sample is displayed (***i-iii***). **b**, simulated reflectance, zoomed in into the experimentally investigated spectral range. **c**, simulated UCPL$_{rel}$ for the maximum design wavelengths measured. The maximum UCPL$_{rel}$ shifts along with the photonic band edge towards smaller wavelengths at higher angles. **d**, simulated UCPL$_{rel}$ for the minimum design wavelengths measured. **e**, measured UCPL$_{rel}$ on three different experimentally investigated Bragg structure designs. The measured range of design wavelengths on each sample is indicated in the graph. The trend for each sample in comparison to the simulation of the maximum (**c**) and minimum (**d**) design wavelengths measured, is in good agreement. Efficient incoupling of light is possible out of a large angle range, up to about 30° (for design ***ii)***.

Supplementary Note 5: Literature Overview

		opal PCs	opal PCs	opal PCs
Publication		Shi, Y. *et al.* Upconversion fluorescence enhancement of $NaYF_4$:Yb/Re nanoparticles by coupling with SiO_2 opal photonic crystals. *J. Mater. Sci.* **54,** 8461–8471 (2019)	Niu, W. B. *et al.* 3-Dimensional photonic crystal surface enhanced upconversion emission for improved near-infrared photoresponse. *Nanoscale* **6,** 817–824 (2014)	Yin, Z. *et al.* Remarkable enhancement of upconversion fluorescence and confocal imaging of PMMA Opal/$NaYF_4$:Yb^{3+}, Tm^{3+}/Er^{3+} nanocrystals. *ChemComm* **49,** 3781–3783 (2013)
Photonic structure	Photonic Structure	SiO2 opal	3D PC of monodisperse carboxylate-modified polystyrene spheres	PMMA opal PC
	Number of designs investigated	2	3	5
UC material	UC material	$NaYF_4$:Yb/Er and $NaYF_4$:Yb/Tm	Yb/Er and Yb/Tm co-doped $NaYF_4$	$NaYF_4$:Yb^{3+}, Tm^{3+}/Er^{3+}
	Form of UC material	UCNPs	UCNPs	UCNPs
	Amount of UC material in structure	~1 µm thin layer on top of opal PC	~ 200 nm thin layer on top of opal PC	one thin layer in spaces around opal colloids on top layer, thickness not reported
	Amount of UC material limited by photonic structure design	yes	yes	yes
Reference	Reference structure	Thin UC nanoparticle film on glass substrate	Thin UC nanoparticle film on glass substrate	Thin UC nanoparticle film on glass substrate
Excitation	Irradiance	-	4 W cm^{-2}	-
	Power	400 mW		5 mW
	Wavelength	980 nm	980 nm	980 nm
	Angle (to surface normal)	-	0°	65° - 90° (0° - 25° off surface plane)
Detection	Angle (to surface normal)	-	-	180° transmission
UC enhancement	Wavelength/emission	450 nm / 541 nm	540 nm / 650 nm	"overall UC" of emissions between 400 nm and 800 nm
	Enhancement factor	34 /23	30 / 30	30
	Spectral width	-	-	-
	Angle width	-	-	-
Simulation	Reflectance/transmittance to determine position of bandgap	yes	yes	yes
	Electric field intensity	-	-	-
	Local density of optical states	-	-	-
	Upconversion dynamics	-	-	-

Supplementary Table 1: Overview of design, experimental parameters and simulations of photonic structures for upconversion (UC) enhancement. A selection of reports on opal photonic crystals (PCs). (Further abbreviations: upconverter nanoparticles (UCNPs)).

		Inverse opal PCs	Inverse opal PCs	2D PCs
**Publication**		Xu, S. *et al.* $NaYF_4$:Yb,Tm nanocrystals and TiO_2 inverse opal composite films: a novel device for upconversion enhancement and solid-based sensing of avidin. *Nanoscale* **6,** 5859–5870 (2014)	Zhang, F., Deng, Y., Shi, Y., Zhang, R. & Zhao, D. Photoluminescence modification in upconversion rare-earth fluoridenanocrystal array constructed photonic crystals. *J. Mater. Chem.* **20,** 3895–3900 (2010)	Wang, H. *et al.* Remarkable enhancement of upconversion luminescence on 2-D anodic aluminum oxide photonic crystals. *Nanoscale* **8,** 10004–10009 (2016)
**Photonic structure**	**Photonic Structure**	TiO2 inverse opal PCs	polystyrene inverse opal PCs	anodic aluminum oxides two-dimensional photonic crystal
	**Number of designs investigated**	5	3	~25
**UC material**	**UC material**	$NaYF_4:Yb^{3+},Tm^{3+}(Er^{3+})$	$NaYF_4:Yb^{3+}/Er^{3+}$	$NaYF_4:Yb^{3+}\ Er^{3+}$
	**Form of UC material**	UCNPs	UCNPs	UCNPs
	**Amount of UC material in structure**	UCNPs are embedded in the voids of TiO2 IOPCs, thickness not reported	inside the voids of the IOPC<br>1 cm pathlength sample investigated in cuvette	1.3 µm thin film on top of 2D PC
	**Amount of UC material limited by photonic structure design**	no	no	yes
**Reference**	**Reference structure**	Thin UC nanoparticle film on glass substrate	UCNPs in cuvette	Thin UC nanoparticle film on glass substrate
**Excitation**	**Irradiance**	48 mW mm^{-2}	-	33 W cm^{-2}
	**Power**	0.1 - 0.9 W	800 mW	
	**Wavelength**	980 nm	978 nm	980 nm
	**Angle (to surface normal)**	0°	-	0°
**Detection**	**Angle (to surface normal)**	180° transmission	-	~ 45°
**UC enhancement**	**Wavelength/emission**	"overall UC" of emissions between 300 nm and 800 nm	515 nm, 565 nm, 640 nm, 675 nm	"overall UC" of emissions of red and green / green / red
	**Enhancement factor**	43 (decreasing for higher power)	4.6 (same for each emission reported)	65 / 50 / 130
	**Spectral width**	-	-	-
	**Angle width**	-	-	-
**Simulation**	**Reflectance/transmittance to determine position of bandgap**		yes	yes
	**Electric field intensity**	-	-	yes
	**Local density of optical states**	-	-	-
	**Upconversion dynamics**	-	-	-

Supplementary Table 2: Overview of design, experimental parameters and simulations of photonic structures for upconversion (UC) enhancement. A selection of reports on inverse opal photonic crystals (PCs) and 2D PCs. (Further abbreviations: upconverter nanoparticles (UCNPs)).

		Waveguides	Cavities	Cavities
**Publication**		Lin, J. H. *et al.* Giant Enhancement of Upconversion Fluorescence of $NaYF_4:Yb^{3+},Tm^{3+}$ Nanocrystals with Resonant Waveguide Grating Substrate. *ACS Photonics* **2,** 530–536 (2015)	Rojas-Hernandez, R. E., Santos, L. F. & Almeida, R. M. Photonic crystal assisted up-converter based on Tb^{3+} / Yb^{3+} - Doped aluminosilicate glass. *Opt. Mater.* **83,** 61–67 (2018)	Yang, J., Li, A.-H., Chen, C. & Sun, Z. Cavity controlled upconversion luminescence in Ag-capped $NaYF_4$:Yb,Er micron rod. *J. Lumin.* **187,** 466–470 (2017)
**Photonic structure**	**Photonic Structure**	waveguide structure	microcavity structure consisting of 21 layers: two Bragg reflectors of alternating TiO2 and Tb^{3+}/Yb^{3+} - doped aluminosilicate glass, seperated by Tb^{3+}/Yb^{3+} - doped aluminosilicate glass defect layer	Ag-capped β-NaYF4:Yb,Er micron rods on a PDMS cavity
	**Number of designs investigated**	1	9	32
**UC material**	**UC material**	$NaYF_4:Yb^{3+},Tm^{3+}$	Tb^{3+}/Yb^{3+}	β-$NaYF_4$:Yb,Er
	**Form of UC material**	UCNPs	UC doped aluminosilicate glass	UC micron rods
	**Amount of UC material in structure**	~200 nm thin layer on top of waveguide structure	11 layers with a summed up thickness of ~ 1 µm	micron rods length 8 µm , diamter 1.8 µm
	**Amount of UC material limited by photonic structure design**	yes	no	yes
**Reference**	**Reference structure**	Nonpatterned area of the sample	Layer stack of only the Tb3+/Yb3+-doped aluminosilicate layers. Seperate reference for each microcavity design	i) Bare rods on glass and ii) 200 nm Ag film on glass and bare rods on top
**Excitation**	**Irradiance**	65 W cm^{-2} (scanned ~10-70 W cm^{-2})	-	500 W cm^{-2}
	**Power**		2 W (scan 1 W to 4 W)	
	**Wavelength**	976 nm	975 nm	975 nm
	**Angle (to surface normal)**	0° - 50°		0°
**Detection**	**Angle (to surface normal)**	0° - 50°	one distinct detection angle (not given which)	0° (using beam splitting)
**UC enhancement**	**Wavelength/emission**	450nm / 480 nm / 650 nm	green	to reference *i* Including statistics of red and green / max for green / max for red / to refence *ii*
	**Enhancement factor**	$6.8*10^4$ / $8.8*10^4$ / $1.6*10^4$	25	4 / 5.2 / 5.0 / 1.8
	**Spectral width**	Enhancement occurs only at very specific excitation wavelengths	-	-
	**Angle width**	Extremely narrow excitation angle range of maximally 0.75° (half angle) is enhanced. UC enhancement on center at 31.5° is 10^4, off center at 30.75° it drops by 3 orders of magnitude to 1.4.	-	-
**Simulation**	**Reflectance/transmittance to determine position of bandgap**	yes	yes	yes
	**Electric field intensity**	yes	-	yes
	**Local density of optical states**	-	-	FDTD simulations of electric dipole emission and Purcell factor
	**Upconversion dynamics**	-	-	-

Supplementary Table 3: Overview of design, experimental parameters and simulations of photonic structures for upconversion (UC) enhancement. A selection of reports on waveguide and cavity structures. (Further abbreviations: upconverter nanoparticles (UCNPs)).

		Multilayer stacks	Multilayer stacks
**Publication**		Johnson, C. M., Reece, P. J. & Conibeer, G. J. Theoretical and experimental evaluation of silicon photonic structures for enhanced erbium up-conversion luminescence. *Sol. Energy Mater. Sol. Cells* **112,** 168–181 (2013)	Hofmann et al, Upconversion enhancement in 1D-photonic crystals: bringing together theory and experiment, manuscript under consideration (2020)
**Photonic structure**	**Photonic Structure**	multilayer stack of ~60 layers of Er^{3+}-doped porous silicon with alternating refractive index (both, high and low refractive index layers are doped)	Bragg structure made of 4 active layers of PMMA with embedded UCNPs and 5 surrounding TiO2 layers
	**Number of designs investigated**	1	40
**UC material**	**UC material**	Er^{3+}	$NaYF_4$:25%Er^{3+}
	**Form of UC material**	UC doped porous silicon	UCNPs
	**Amount of UC material in structure**	thin layers with ~ 15 µm summed up thickness	~1.2 µm summed up thickness of all UC layers
	**Amount of UC material limited by photonic structure design**	no	no
**Reference**	**Reference structure**	None. The enhancement is calculated relative to the lowest measured UC emission at 38°	Layer stack of only the UC layers on glass to gain the same total thickness as the UC layers in each Bragg structure
**Excitation**	**Irradiance**	-	max at 1.48 W cm^{-2} (scan ~ 0.18 W cm^{-2} - 1.5 W cm^{-2})
	**Power**	200 mW	1 mW - 10 mW
	**Wavelength**	1550 nm	1523 nm for max UC enh.<br>1500 nm - 1560 scan
	**Angle (to surface normal)**	max UC enhancement at 34°, scanned: 21° - 38°	4° for max, and scanned 0° - 75°
**Detection**	**Angle (to surface normal)**	0°	Integrating sphere
**UC enhancement**	**Wavelength/emission**	980 nm / green	984 nm / 814 nm
	**Enhancement factor**	5 / 26.5	max 4.1 (mean 2.4) / ~5
	**Spectral width**	-	~ 60 nm
	**Angle width**	~ 4° (full angle)<br>max UC enhancement at 34° incident angle, dropping by about a factor of 5 within 2° to lower and higher angles)	~ 30° half angle
**Simulation**	**Reflectance/transmittance to determine position of bandgap**	yes	yes
	**Electric field intensity**	-	yes
	**Local density of optical states**	-	MIT Photonic bands simulation and histogramming method to determine local density of optical states
	**Upconversion dynamics**	-	rate equation model of UC dynamics including the impact of photonic effects and production accuracy of the photonic structure

Supplementary Table 4: Overview of design, experimental parameters and simulations of photonic structures for upconversion (UC) enhancement. A selection of reports on multilayer stacks. (Further abbreviations: upconverter nanoparticles (UCNPs)).

REFERENCES

1. Fischer, S., Steinkemper, H., Löper, P., Hermle, M. & Goldschmidt, J. C. Modeling upconversion of erbium doped microcrystals based on experimentally determined Einstein coefficients. *J. Appl. Phys.* **111,** 13109 (2012).
2. Walsh, C. B. & Franses, E. I. Ultrathin PMMA films spin-coated from toluene solutions. *Thin Solid Films* **429,** 71–76 (2003).
3. J.A. Woollam Company. CompleteEASE Software Manual.
4. Yang, H. G. *et al.* Anatase TiO_2 single crystals with a large percentage of reactive facets. *Nature* **453,** 638–641 (2008).
5. Pore, V. *et al.* Atomic Layer Deposition of Photocatalytic TiO2 Thin Films from Titanium Tetramethoxide and Water. *Chem. Vap. Deposition* **10,** 143–148 (2004).
6. Lee, C.-S., Kim, J., Son, J. Y., Choi, W. & Kim, H. Photocatalytic functional coatings of TiO_2 thin films on polymer substrate by plasma enhanced atomic layer deposition. *Appl. Catal. B: Environmental* **91,** 628–633 (2009).
7. Ma, X. Y. *et al.* Fabrication of uniform anatase TiO_2 particles exposed by {001} facets. *ChemComm* **46,** 6608–6610 (2010).
8. Kartini, I. *et al.* Hydrothermal seeded synthesis of mesoporous titania for application in dye-sensitised solar cells (DSSCs). *J. Mater. Chem.* **14,** 2917 (2004).
9. Hofmann, C. L. M. *et al.* Enhanced upconversion in one-dimensional photonic crystals. A simulation-based assessment within realistic material and fabrication constraints. *Opt. Express* **26,** 7537 (2018).
